# *De novo* diagnostics of patients with intellectual disability

**DOI:** 10.1186/1753-6561-6-S6-O9

**Published:** 2012-10-01

**Authors:** Joris A Veltman

**Affiliations:** 1 Department of Human Genetics, Radboud University Nijmegen Medical Centre, PO Box 9101, 6500 HB Nijmegen, The Netherlands

## 

Germline coding *de novo* mutations (SNVs, indels as well as CNVs) are an important cause of moderate to severe forms of intellectual disability (ID) and associated syndromes. Exome sequencing now allows us to reliably identify these mutations using a single genomic test, and we have recently implemented exome sequencing in the diagnostic follow-up of these patients.

In this presentation, I will first discuss the role of *de novo* mutations in genetic disease and the associated risk factors such as local genomic structure and paternal age. Next, I will describe our recent work using a diagnostic family-based exome sequencing approach to test this *de novo* mutation hypothesis in 100 patients with unexplained ID, as well as targeted follow-up studies of several candidate ID genes in 750 additional patients. A total of 79 unique coding *de novo* mutations were identified and validated in 52 patients. Damaging *de novo* (n = 10) as well as X-linked maternally-inherited (n = 3) mutations were detected in known ID genes, resulting in a minimal diagnostic yield of 13% in this cohort. In addition, potentially causative *de novo* mutations in novel candidate ID genes were detected in 22 patients. For three of these candidate genes, recurrent *de novo* mutations were identified in patients with similar phenotypes, confirming that they are true ID genes. To further expand the possibilities of exome sequencing for mutation detection, we have recently implemented automatic CNV detection on exome data, and compared its performance to that of high-resolution genomic microarrays. This analysis shows that exome sequencing can reliably detect the large majority of pathogenic *de novo* CNVs, responsible for approximately 15% of ID.

In conclusion, *de novo* mutations therefore represent an important cause of ID, and exome sequencing is an effective diagnostic strategy for their detection.
